# Statistics of Homœopathy in New York

**Published:** 1850-11

**Authors:** 


*Statistics of Homœopathy in New York.*—In our last No. we published statistics of Homœopathy in Great Britain. In a late No. of the Medical Gazette we find a list of the Homœopathic practitioners in the city of New York. The whole number officially announced by the *Hahnemann Academy of Medicine* is *thirty-five!* The number of regular practitioners in the city of New York is 875. The numerical proportion of Homœopathic practitioners to the Medical Faculty in that city, therefore, is *one-twenty-fifth;* and the proportion to the population is *one* for every 14,000! The letters of M. D. are affixed to all the names announced by the *Hahnemann Academy*, but the Editor of the Gazette surmises that in most instances the title is assumed. Empirics of all complexions are apt to testify their real estimation of the respectability of the Profession they affect to despise, by stealing its insignia.

